# Melanoma Cell Resistance to Vemurafenib Modifies Inter-Cellular Communication Signals

**DOI:** 10.3390/biomedicines9010079

**Published:** 2021-01-15

**Authors:** Claudio Tabolacci, Martina Cordella, Sabrina Mariotti, Stefania Rossi, Cinzia Senatore, Carla Lintas, Lauretta Levati, Daniela D’Arcangelo, Antonio Facchiano, Stefania D’Atri, Roberto Nisini, Francesco Facchiano

**Affiliations:** 1 Department of Oncology and Molecular Medicine, Istituto Superiore di Sanità, Viale Regina Elena 299, 00161 Rome, Italy; martina.cord87@gmail.com (M.C.); stefania.rossi@iss.it (S.R.); cinzia.senatore@gmail.com (C.S.); 2 Department of Infectious Diseases, Istituto Superiore di Sanità, 00161 Rome, Italy; sabrina.mariotti@iss.it (S.M.); roberto.nisini@iss.it (R.N.); 3 Department of Experimental Medicine, University Campus Bio-Medico, 00128 Rome, Italy; c.lintas@unicampus.it; 4 Laboratory of Molecular Oncology, IDI-IRCCS, 00167 Rome, Italy; l.levati@idi.it (L.L.); d.darcangelo@idi.it (D.D.); a.facchiano@idi.it (A.F.); s.datri@idi.it (S.D.)

**Keywords:** melanoma, BRAF inhibitors resistance, secretory signals, inflammation, basigin

## Abstract

The therapeutic success of BRAF inhibitors (BRAFi) and MEK inhibitors (MEKi) in BRAF-mutant melanoma is limited by the emergence of drug resistance, and several lines of evidence suggest that changes in the tumor microenvironment can play a pivotal role in acquired resistance. The present study focused on secretome profiling of melanoma cells sensitive or resistant to the BRAFi vemurafenib. Proteomic and cytokine/chemokine secretion analyses were performed in order to better understand the interplay between vemurafenib-resistant melanoma cells and the tumor microenvironment. We found that vemurafenib-resistant melanoma cells can influence dendritic cell (DC) maturation by modulating their activation and cytokine production. In particular, human DCs exposed to conditioned medium (CM) from vemurafenib-resistant melanoma cells produced higher levels of pro-inflammatory cytokines—that potentially facilitate melanoma growth—than DCs exposed to CM derived from parental drug-sensitive cells. Bioinformatic analysis performed on proteins identified by mass spectrometry in the culture medium from vemurafenib-sensitive and vemurafenib-resistant melanoma cells suggests a possible involvement of the proteasome pathway. Moreover, our data confirm that BRAFi-resistant cells display a more aggressive phenotype compared to parental ones, with a significantly increased production of interferon-γ, interleukin-8, vascular-endothelial growth factor, CD147/basigin, and metalloproteinase 2 (MMP-2). Plasma levels of CD147/basigin and MMP-2 were also measured before the start of therapy and at disease progression in a small group of melanoma patients treated with vemurafenib or vemurafenib plus cobimetinib. A significant increment in CD147/basigin and MMP-2 was observed in all patients at the time of treatment failure, strengthening the hypothesis that CD147/basigin might play a role in BRAFi resistance.

## 1. Introduction

Melanoma is an aggressive form of skin cancer characterized by poor prognosis in the late stages. However, in recent years, outcomes of melanoma patients have greatly improved due to new therapies specifically targeting oncogenic driver mutations or immune checkpoints [1]. Oncogenic mutations in BRAF are present in up to 50% of melanomas, the most frequent being a valine to glutamic acid at position 600 (V600E), which constitutively activates the BRAF/MEK/ERK-signaling pathway, transmitting constant cell growth signals [2].

The BRAF inhibitors (BRAFi) vemurafenib and dabrafenib, the MEK inhibitor (MEKi) trametinib, and combinations of BRAFi and MEKi (dabrafenib + trametinib, vemurafenib + cobimetinib, encorafenib + binimetinib) are presently approved as first line therapies for patients with unresectable or metastatic melanoma harboring BRAF^V600E^ (vemurafenib, dabrafenib) or BRAF^V600E/K^ (trametinib, combined therapies) [3,4,5]. Unfortunately, although most patients experience a remarkable initial response to BRAFi and MEKi, the long-term efficacy of therapy is limited by the development of secondary drug resistance in the majority of patients [6].

Several mechanisms are considered responsible for acquired resistance to targeted therapy, including secondary mutations, bypass signaling and activation of other compensatory downstream effectors, modifications of the tumor microenvironment (TME), and cross talk with the immune system [7,8]. In particular, melanoma is characterized by a remarkable metabolic plasticity that leads to the development of resistance mechanisms through metabolic interactions between tumor cells and the TME [9]. Moreover, it has been demonstrated that treatment with BRAFi/MEKi can induce TME modifications through autocrine and paracrine effects [10]. In fact, melanoma is an immunogenic cancer that escapes immune surveillance through the production of specific cytokines and growth factors in the TME [11].

Dendritic cells (DCs) are professional antigen-presenting cells physiologically present in tissues, and they exert a pivotal role in immune surveillance through the regulation of both innate and adaptive immunity. Appropriate stimuli—such as pathogen-associated molecular patterns (PAMPS) able to stimulate Toll-like receptors or cytokines—cause the switch from immature to mature DCs with a phenotype characterized by increased expression of both stimulatory and co-stimulatory molecules, the acquisition of activation markers, and the active secretion of cytokines and/or chemokines [12]. DCs are a critical component of antitumor immunity, being potent inducers of T cell responses. On the other hand, defects in DC maturation and function have also been described in several types of cancer, including melanoma [13], suggesting that, under certain circumstances, DCs can contribute to immune suppression and tumor progression [14]. A better knowledge of whether and how the secretory pathways of melanoma cells with acquired resistance to BRAFi lead to modifications of the TME and affect DCs is essential for developing new therapeutic approaches aimed at increasing drug sensitivity and overcoming the emergence of secondary resistance.

In this study, the capacity of conditioned media from vemurafenib-resistant melanoma cells to modulate or interfere with DC activation was studied. Moreover, proteins released in the medium by melanoma cells showing acquired resistance were identified to better clarify their interplay with the TME.

## 2. Materials and Methods

### 2.1. Chemicals and Reagents

Roswell Park Memorial Institute medium (RPMI-1640), phosphate-buffered saline without Ca^++^ and Mg^++^ (PBS), glutamine, penicillin (10,000 UI/mL), and streptomycin (10,000 μg/mL) were from Eurobio Laboratoires (Le Ulis Cedex, France). Fetal calf serum (FCS) was from HyClone (South Logan, UT, USA). All solvents were purchased from Mallinckrodt Baker (Milan, Italy). Dimethyl sulfoxide (DMSO), lipopolysaccharide (LPS) of *Escherichia coli*, and all other reagents were from Sigma Chemicals (St. Louis, MO, USA). Vemurafenib (from Selleck Chemicals, Houston, TX, USA) and bortezomib (from Santa Cruz Biotechnology, Santa Cruz, CA, USA) were dissolved in DMSO. Matrigel (MG) was from Becton Dickinson Bioscience (Franklin Lakes, NJ, USA). Granulocyte-macrophages colony stimulating factor (GM-CSF) was from Sandoz (Basel, Switzerland), and recombinant human interleukin (IL)-4 was purchased from R&D Systems (Minneapolis, MN, USA).

### 2.2. Cell Culture and Generation of Vemurafenib-Resistant Cell Lines

The BRAF-mutant (V600E) human melanoma cell line SK-MEL-28 [15] was obtained from the American Type Culture Collection (ATCC). Cells were cultured in RPMI-1640, supplemented with 10% FCS, 0.05% L-glutamine, penicillin (100 U/mL), and streptomycin (100 μg/mL) and maintained at 37 °C in a 5% CO_2_ humidified atmosphere. Vemurafenib-resistant (VR) variants (namely VR2 and VR3) were derived from the original parental cell line according to a published procedure [16]. Briefly, human melanoma cells were initially treated with 20 µM vemurafenib and then cultured in complete medium containing 5 µM vemurafenib for at least 3 months before they were used for the subsequent studies. Several resistant subcultures were obtained, and these cells were further propagated in growth medium containing 2 µM vemurafenib. Resistant clones were cultured for one cell cycle in the absence of vemurafenib before each experiment.

Evaluation of cell growth in the presence of vemurafenib was achieved by a colori-metric sulphorhodamine B (SRB) assay (cell density determination based on cellular pro-tein content), as previously described [17]. Briefly, melanoma cells (4 × 10^3^ cells/well) were seeded and grown in 96-well plates for 24 h and then exposed to different concentrations of vemurafenib for 72 h. Subsequently, cells were fixed with 50% trichloroacetic acid and stained with 0.2% SRB solution. Cell proliferation was determined by spectrophotometric quantification (540 nm wavelength).

### 2.3. Expression Levels of Cytokines/Chemokines in Conditioned Medium

Cytokine/chemokine quantification in cell cultures was achieved by xMAP technology through a Luminex platform (Bio-Rad Laboratories, Hercules, CA, USA) equipped with a magnetic washer workstation according to the manufacturer’s protocol. Melanoma cell lines were cultured in complete medium for 24 h. Then cells were washed in PBS, and fresh complete medium was applied. The conditioned medium (CM) was collected 72 h later and stored at −20 °C until needed. Samples were analyzed (using a Bio-Plex Pro human cytokine multiplex assay) for IL-1β, IL-4, IL-6, IL-8, IL-10, IL-12, tumor necrosis factor(TNF)-α, interferon(IFN)-γ, Eotaxin/CCL11, basic fibroblast growth factor (bFGF), GM-CSF, granulocyte-colony stimulating factor (G-CSF), monocyte chemoattractant protein-1 (MCP-1/CCL2), RANTES/CCL5 (Regulated on Activation, Normal T-expressed and Secreted), macrophage inflammatory protein 1 alpha (MIP-1α/CCL3), MIP-1β/CCL4, IFN-γ-inducible protein 10 (IP-10/CXCL10), and vascular endothelial growth factor (VEGF). The quantification was carried out with a Bio-Plex array reader (Bio-Plex 200 System) and Bio-Plex Manager (Version 6.1 Bio-Rad Laboratories, Hercules, CA, USA) software.

### 2.4. Generation of Dendritic Cells and Swap Experiments

Human DCs were differentiated from monocytes isolated from human peripheral blood mononuclear cells of healthy donors according to published methods [18]. Briefly, cells were isolated by Ficoll density gradient, and then monocytes were positively sorted using anti-CD14-labeled magnetic beads (MACS, Miltenyi Biotech, Germany) according to the manufacturer’s instructions. The sorted cells, obtained from two different healthy donors, were cultured for 5 days in complete medium supplemented with 25 ng/mL GM-CSF and 1000 U/mL IL-4 at a cell density of 4 × 10^5^ cells/mL in 6-well plates (3 mL/well) to induce their differentiation into DCs.

For medium swap experiments at the 5th day of culture, DCs were collected, washed, and plated overnight at 5 × 10^5^ cells/mL in 24-well plates (1 mL/well) in complete medium containing melanoma CM previously analyzed for cytokines/chemokines content (20% vol/vol). To control maturation, 0.2 µg/mL LPS was added overnight. When required, melanoma CM was added overnight together with LPS.

### 2.5. FACS Analysis

DCs were collected and stained for 30 min at 4 °C with the following mAbs: anti-human HLA class I, HLA-DR class II, CD80, CD83, and CD86 or appropriate isotype controls (all from BD Pharmingen, San Diego, CA, USA) according to the manufacturer’s instructions. Then, stained cells were analyzed using a Beckman Coulter Gallios flow cytometer equipped with three lasers and Kaluza Software (Beckman Coulter), acquiring 2 × 10^4^ events gated according to DC forward and size scatters.

### 2.6. Collection of Cell Line Secretome, Mass Spectrometry, and Protein Identification

Melanoma cells (SK-MEL-28 and SK-MEL-28-VR2) were cultured in standard conditions until they reached 70% confluence. Culture medium was then removed, and cells were washed three times with PBS to eliminate residual FCS. To collect the secretome, cells were incubated with serum-free medium for 24 h at 37 °C. For each cell type, serum-free conditioned media (SF-CM) from three different flasks were pooled and centrifuged to remove cellular debris. SF-CM were then concentrated (10-fold) using MWCO of 3 kDa Ultra centrifugal filters (Amicon Millipore, Billerica, MA, USA) at 4 °C.

To optimize their proteomic identification, proteins (10 µg) from concentrated SF-CM were denatured and electrophoretically separated as described [19]. Briefly, the total gel lanes were cut, and proteins were reduced, alkylated, and digested overnight with bovine trypsin sequencing grade (Roche Applied Science, Monza, Italy). The peptide mixtures were then analyzed by nano-reversed-phase liquid chromatography tandem mass spectrometry (RP-LC-MS/MS), and proteins were identified as described [19,20].

### 2.7. Bioinformatics Analysis

Differentially expressed proteins identified by proteomic analysis were further analyzed by the Database for Annotation, Visualization, and Integrated Discovery (version 6.8, DAVID) software (http://david.abcc.ncifcrf.gov/) [21]. DAVID functional annotation cluster analysis was performed on the list of proteins of SF-CM from SK-MEL-28 and SK-MEL-28-VR2 identified by LC-MS/MS analysis. Terms with a *p*-value ≤ 0.05 were selected for DAVID analysis. The gene ontology (GO) terms of cellular component (GOTERM_CC_FAT), molecular function (GOTERM_MF_DIRECT), biological processes (GOTERM_BP_DIRECT), and UP_KEYWORDS in the functional categories section in DAVID were used. To obtain GO (significant *q*-value threshold level of <0.05) of differentially expressed proteins, we also used the GOnet database (https://tools.dice-database.org/GOnet/) [22]. Proteins were categorized according to GO molecular function. Functional protein–protein interactions were also analyzed using STRING software, version 10.5 (http://string-db.org). The interaction networks were obtained on the basis of confidence scores (threshold score 0.4 with no more than 5 interactors) as described [23].

### 2.8. Evaluation of Melanoma Cell Sensitivity to Bortezomib, Adhesion Assay, and Determination of CD147/Basigin and MMP-2

For proliferation studies, cells (8 × 10^4^) were seeded and grown for 24 h in 6-well plates. Thereafter, cells were treated with bortezomib (0, 10, 20, and 40 nM) for 24 h. Cells were then harvested and counted with a Neubauer modified chamber.

The adhesion assay was performed on 24-well plates coated with MG (50 μg/well). After MG polymerization, cells were seeded at a density of 1 × 10^6^ cells/mL, followed by incubation at 37 °C for 1 h. The adherent cells were detached with trypsin/EDTA and counted. Attachment to MG was expressed as the percentage of cells adhered, and the percentage of parental cell line was taken as 100%.

Surface expression of CD147/basigin (or extracellular matrix metalloproteinase inducer, EMMPRIN) in melanoma cells was determined by flow cytometry using a FITC-conjugated anti-CD147 antibody (BD Pharmingen, kindly provided by Dr. Elvira Pelosi, Istituto Superiore di Sanità, Rome, Italy), as previously described [20]. CD147/basigin and matrix metalloproteinase 2 (MMP-2) in CM were quantified using a human magnetic Luminex assay (R&D Systems, Minneapolis, MN, USA) according to manufacturer’s instructions.

### 2.9. Patients

Plasma levels of CD147/basigin and MMP-2 were determined (as described in the previous paragraph) also in 5 patients with BRAF^V600^-mutant metastatic cutaneous melanoma treated with either vemurafenib or vemurafenib plus cobimetinib at Istituto Dermopatico dell’Immacolata, IDI-IRCCS; from such patients, peripheral blood samples were collected both before the start of therapy and at disease progression. Baseline evaluation included medical history, physical examination, and radiologic tumor assessment with computer tomography (CT) or positron emission tomography scans. Vemurafenib (Zelboraf) was given at a dose of 960 mg/bid, and vemurafenib plus cobimetinib (Cotellic) at a dose of 960 mg/bid and 60 mg/qd, respectively, for three weeks with one week of break. All patients underwent physical examination and assessment of biochemical parameters monthly, whereas tumor response was determined with CT every three months. Tumor response was classified according to RECIST 1.1 criteria. Time-to-treatment-failure (TTF) was defined as the time from the start of therapy to the first observation of disease progression per RECIST 1.1. The study was conducted in accordance with good clinical practice guidelines and the Declaration of Helsinki. The study was approved by the IDI-IRCCS Ethics Committee (ID #407/1, 2013 and #407/2, 2016), and a written informed consent was obtained from the patients.

### 2.10. Plasma Preparation

Blood was collected into BD vacutainer tubes (#367704, BD Biosciences, Plymouth, UK) and centrifuged at 1200× *g* for 10 min at 4 °C. Plasma was collected and centrifuged again at 1200× *g* for 10 min at 4 C°, aliquoted, and stored at −80 °C until use.

### 2.11. Statistical Analysis

Results are expressed as means of three independent experiments ± standard deviations (SDs). The statistical significance of differences was determined by two-tailed *t*-tests; the significance threshold was set at *p* ≤ 0.01. The analysis of the plasma CD147/basigin and MMP-2 expression levels was carried out with Mann–Whitney tests; the significance threshold was set at *p* < 0.05.

## 3. Results

### 3.1. Identification of Cytokines in Conditioned Media from Vemurafenib-Resistant Cells

**Figure 1:**
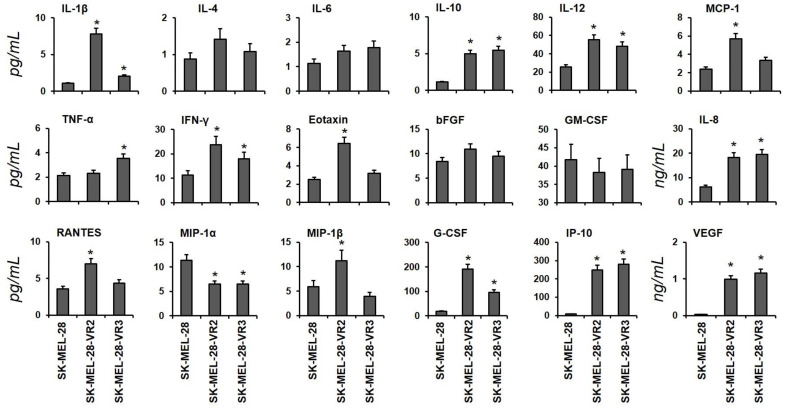
Analysis of cytokines and chemokines secreted by SK-MEL-28 (sensitive, parental) and vemurafenib-resistant (VR2 and VR3) melanoma cells. Concentrations of the indicated analytes in conditioned media (CM) were quantified by multiplex immunoassay. Results are shown as mean ± SD of triplicate samples (statistical significance versus sensitive cell lines: * *p* < 0.01).

For this study, resistant cell lines (two independent clones, namely VR2 and VR3) were generated by chronic exposure of SK-MEL-28 cells to vemurafenib, and acquired resistance was confirmed by SRB assay (Figure S1). To investigate the secretome of vemurafenib-resistant cells, the cytokine/chemokine expression profiles of VR2 and VR3 cells were analyzed using a multiplex assay. The data, summarized in Figure 1, reveal that several analytes—namely IL-1β, IL-8, IL-10, IL-12, IFN-γ, G-CSF, IP-10, and VEGF—were increased in CM from VR2 and VR3 cells as compared to CM derived from the parental cell line. Other soluble factors, such as MCP-1, Eotaxin, RANTES, and MIP-1β, were increased only in VR2 cells. Moreover, the expression levels of MIP-1α were decreased in VR-clones compared to SK-MEL-28, whereas other cytokine/chemokine levels (i.e., IL-4, IL-6, bFGF, and GM-CSF) were not significantly changed.

### 3.2. Conditioned Media from Resistant Cells Alter Dendritic Cells Phenotype and Cytokines/Chemokine Secretion Pattern

Antitumor immunity is coordinated by both innate and adaptive immunity, and DC activation plays a key role in cancer surveillance. On the basis of the composite profile of cytokines/chemokines in vemurafenib-resistant melanoma CM, we evaluated the influence of tumor-derived factors on DC activation. Human monocyte-derived DCs from two healthy donors were co-cultured with CM derived from SK-MEL-28 cells or VR clones or treated with LPS as positive control. Stimulation of DCs with CM from vemurafenib-resistant clones caused an upregulation of costimulatory molecules (CD80 and CD86) and of the CD83 activation marker and a slight increase of MHC class II presenting molecules (but not class I molecules) as compared to CM-derived from parental cells (Table S1). The expression of these markers in DCs was, however, lower than that achieved upon LPS stimulation (Table S1). Furthermore, upregulation of CD80, CD86, and CD83 on LPS-stimulated DCs was not modified by the addition of melanoma CM, suggesting that at least in our experimental model, the secretome of melanoma cells, either sensitive or resistant to vemurafenib, did not interfere with the activation of DCs mediated by PAMPS (data not shown).

**Figure 2:**
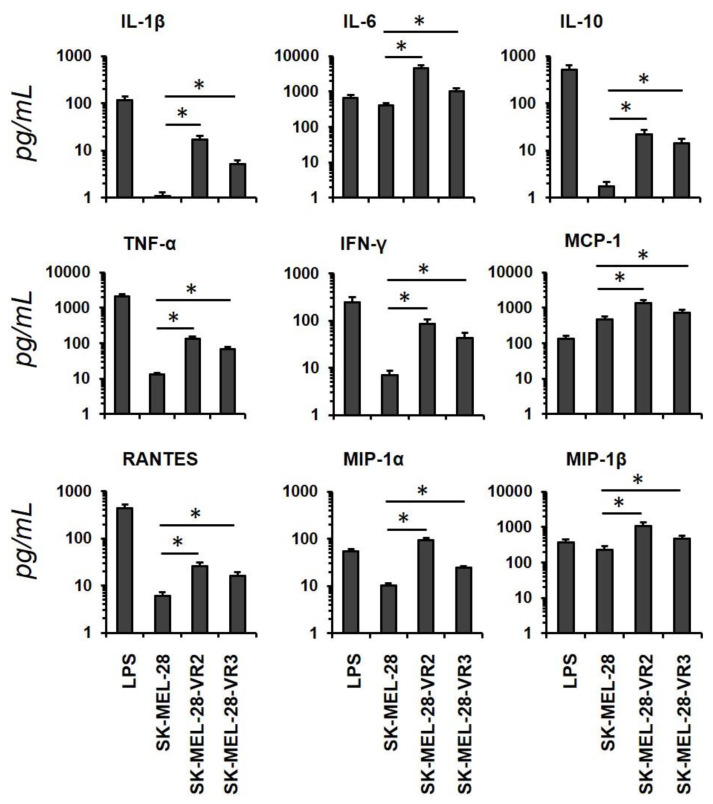
Analysis of cytokines and chemokines secreted by dendritic cells (DCs) incubated overnight with melanoma conditioned media (CM) or lipopolysaccharide (LPS). Concentrations of the indicated proteins in CM were quantified by multiplex immunoassay. Results are shown as mean ± SD of two experiments carried out in duplicate (statistical significance: * *p* < 0.01).

Cytokine/chemokine production in culture supernatants of DCs in the presence of melanoma CM was also investigated. CM derived from the two drug-resistant clones, besides upregulating maturation and activation markers, also increased the secretion of soluble factors (i.e., IL-1β, IL-10, TNF-α, IFN-γ, RANTES) with respect to parental cell line-derived CM. Interestingly, we observed that CM from vemurafenib-resistant cells induced the release of higher amounts of IL-6 and MCP-1 than LPS (Figure 2).

### 3.3. Secretome Profiling of Vemurafenib-Resistant Cells

**Figure 3:**
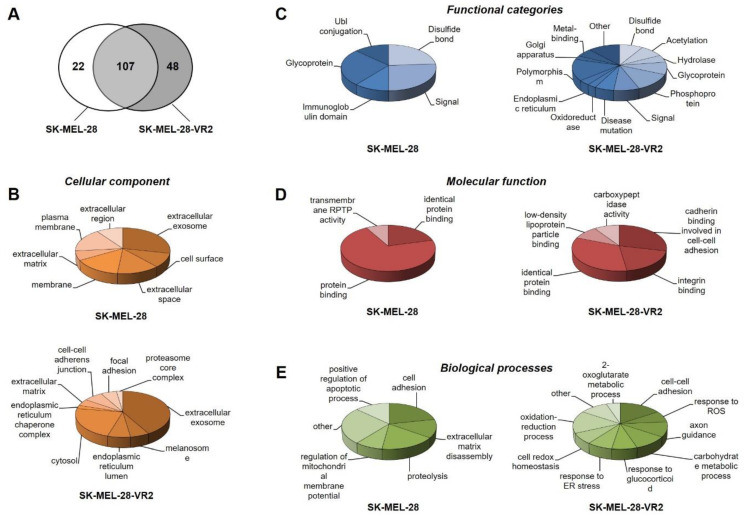
Secretome analysis of sensitive and vemurafenib-resistant melanoma cells. (**A**) The diagram illustrates common and differentially expressed (specific) proteins in SK-MEL-28 (sensitive) and SK-MEL-28-VR2 (vemurafenib-resistant) melanoma cells. The pie charts show Database for Annotation, Visualization, and Integrated Discovery (DAVID) classification of specific proteins based on (**B**) cellular component, (**C**) functional categories, (**D**) molecular functions, and (**E**) biological processes.

In order to identify secreted proteins potentially associated with BRAFi resistance, we further analyzed the secretome of SK-MEL-28 and SK-MEL-28-VR2 cell lines using RP-LC–MS/MS. Under our experimental conditions, a total of 129 and 155 proteins were identified in SK-MEL-28 and SK-MEL-28-VR2 cells, respectively (Table S2), with 107 common proteins (Figure 3A).

The differentially expressed proteins (named “specific” proteins) (Table 1) were classified by DAVID bioinformatic tools. The cellular localization of these proteins confirmed that they can be considered secretory proteins in most cases (Figure 3B). Then, the two groups of specific proteins were classified according to their functional categories (Figure 3C) and molecular functions (Figure 3D). In particular, several SK-MEL-28-VR2 proteins were involved in protein binding (33%), cell–cell adhesion (29%) and integrin binding (19%) phenomena. Finally, according to biological processes classification, the differences between SK-MEL-28 and SK-MEL-28-VR2 secretome were evident, especially for cell adhesion, cell–cell interaction and responses to stress (Figure 3E).

**Figure 4:**
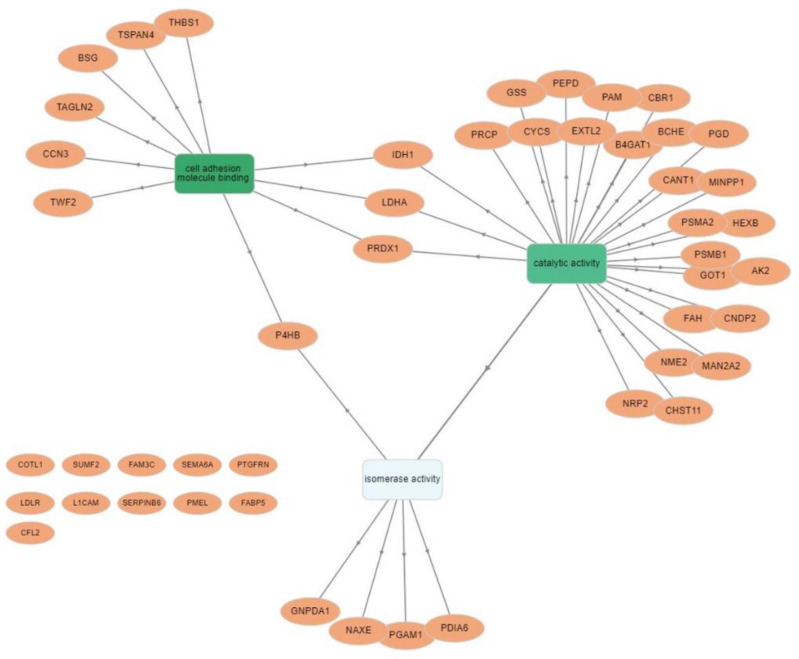
Gene ontology analysis of SK-MEL-28-VR2 cells using GOnet Internet application. Classification of specific proteins was performed according to molecular functions. Arrows indicate direct link between molecular functions according to the GOnet annotation. Round nodes represent proteins; square nodes represent various molecular functions. The darker color of the GOterm square node characterizes higher significance.

Additional cluster analysis was performed on SK-MEL-28-VR2 specific proteins using the GOnet tool. This analysis (molecular function; *p* ≤ 0.0005) highlighted three clear functional clusters related to cell adhesion (higher significance), catalytic activity, and isomerase activity (Figure 4).

### 3.4. Protein–Protein Interaction Network Analysis

**Figure 5:**
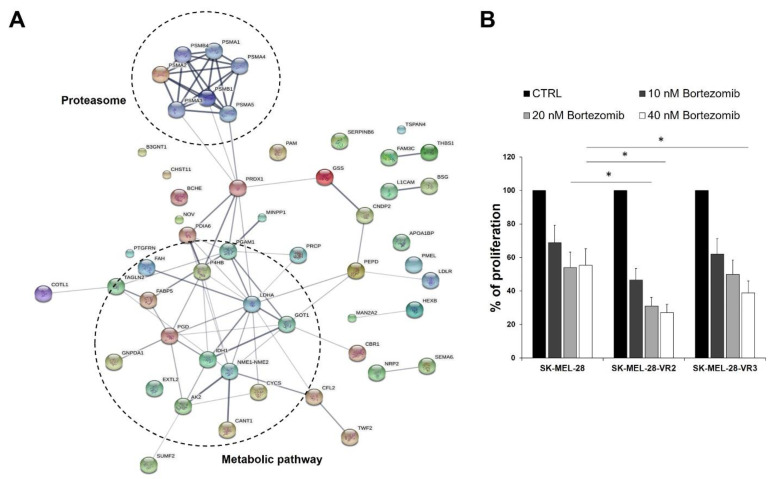
(**A**) The protein–protein interaction network of SK-MEL-28-VR2 differentially expressed proteins as predicted by the STRING software. The links between proteins represent possible interactions (line thickness indicates the strength of association). The two significant pathways were clustered. (**B**) Proliferation rate of melanoma cells left untreated (CTRL) or treated with the proteasome inhibitor bortezomib for 24 h. Data are expressed as % proliferation compared to the CTRL (100%) + SD of three independent experiments (statistical significance: * *p* < 0.01).

With the purpose of highlighting the differences between proteins specifically expressed by SK-MEL-28 and SK-MEL-28-VR2 (Table 1), a protein network was generated. While no significant connectivity was observed in SK-MEL-28 specific proteins (data not shown), a relevant interaction network was found for SK-MEL-28-VR2 cells. In particular, the STRING predictive network analysis depicted strong interaction among proteins involved in proteasome and metabolic pathways (Figure 5A).

Proteasome is a multi-subunit complex with protease activity. The ubiquitin–proteasome pathway represents the most important intracellular system for protein degradation and is involved in several biological processes, such as apoptosis, survival, DNA repair, and antigen presentation [24]. Increased proteasomal degradation of key regulatory proteins promotes cancer cell growth, survival, and chemoresistance [24]; and therefore, the proteasome has been considered a suitable target for antitumor therapy. Among proteasome inhibitors, bortezomib is approved for multiple myeloma and mantle cell lymphoma treatment [25,26]. Based on our protein–protein interaction network analysis indicating a strong interaction among proteins involved in the proteasome pathway in vemurafenib-resistant cells, we sought to investigate whether these cells could be more sensitive to bortezomib than parental cells. As shown in Figure 5B, bortezomib affected melanoma cell growth in a concentration-dependent manner, and both resistant clones (VR2 and VR3) were significantly more sensitive to the drug than parental cells. Therefore, the STRING prediction suggesting increased proteasomal degradation in vemurafenib-resistant cells was supported by a set of cell proliferation experiments showing that the development of secondary resistance to vemurafenib was accompanied by increased sensitivity to proteasome inhibitors.

### 3.5. CD147/Basigin Expression in Vemurafenib-Resistant Cells

**Figure 6:**
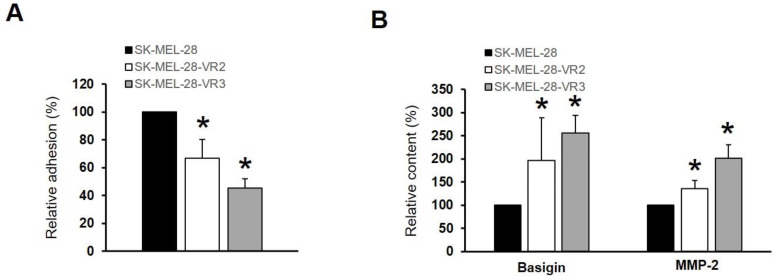
Vemurafenib-resistant cells (VR2 and VR3) showed more disseminative features. (**A**) Decrease of adhesion to extra-cellular matrix facilitated metastatic process. Resistant clones displayed a reduction of adhesion ability. Each point represents the mean ± SD of three different determinations (statistical significance versus control: * *p* < 0.01). (**B**) CD147/basigin and MMP-2 levels in melanoma cell lines as determined by Luminex assay (statistical significance versus control: * *p* < 0.01).

Our data clearly indicate that vemurafenib-resistant cells expressed more proteins related to cell adhesion (Figure 4), cell–cell and cell–matrix interactions (Figure 3), as well as higher levels of cytokines/chemokines able to modify the TME (Figure 1). These features are linked to a more disseminating phenotype of melanoma cells [27]. Accordingly, VR2 and VR3 cells showed a significant decrease of adhesion to reconstituted basement membrane (MG) (Figure 6A). Moreover, proteomic analysis (Table 1) revealed the presence of a transmembrane protein, namely CD147/basigin, in the resistant clone. CD147/basigin is highly expressed in metastatic melanoma and other malignant cells and promotes angiogenesis, tumor progression, and metastasis through the induction of matrix metalloproteinases (MMPs) [28]. Multiplex assay confirmed the presence of CD147/basigin (and MMP-2) in the SF-CM of both resistant clones (Figure 6B), while no differences were observed in CD147/basigin expression on the cell surface (data not shown).

### 3.6. CD147/Basigin and MMP-2 Levels in Melanoma Patients

**Figure 7:**
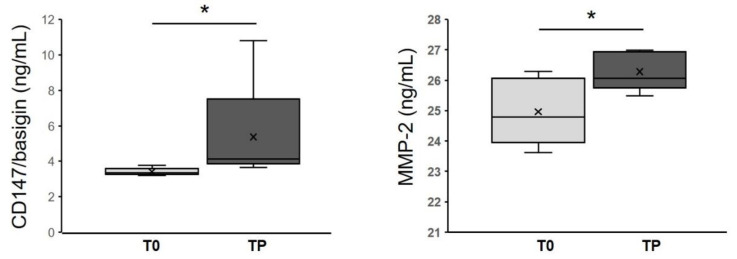
Plasma concentrations of CD147/basigin and MMP-2 in patients with BRAF^V600^-mutant metastatic cutaneous melanoma treated with either vemurafenib or vemurafenib plus cobimetinib. The median plasma levels of the indicated molecules in blood samples collected before the start of therapy (T0) were significantly lower than those collected at disease progression (TP). Statistical significance: * *p* < 0.05.

Based on the finding that vemurafenib-resistant cells secreted higher amounts of CD147/basigin and MMP-2 than drug-sensitive parental cells, a pilot study was carried out to investigate whether increased levels of these two proteins could be also detected in the plasma of melanoma patients developing secondary resistance to vemurafenib or vemurafenib plus cobimetinb therapy. We analyzed plasma from five melanoma patients before the start of therapy and at disease progression, i.e., when resistance appeared. Table 2 summarizes the principal clinical and pathological characteristics of melanoma patients enrolled in this study. Patients ranged in age from 49 to 74 years, with a mean age of 57.6 years. All patients showed a partial response (PR) to therapy, with a range of TTF (time-to-treatment-failure) from 147 to 362 days. As reported in Figure 7, in all the five melanoma patients, CD147/basigin and MMP-2 plasma levels at disease progression (TP) were significantly higher than those measured at the time of enrolment (*p* = 0.015 and *p* = 0.032, respectively). These results reinforce the data obtained in the melanoma cell lines and suggest that upregulation of CD147/basigin and MMP-2 might contribute to the development of secondary resistance to BRAFi and MEKi.

## 4. Discussion

Understanding the interaction between tumor cells and the TME is essential to identifying new therapeutic strategies. Indeed, the cross talk between tumor cells and the TME contributes to tumor progression, metastasis, and response to therapy [29]. In this regard, it has been clearly demonstrated that the TME is able to support proliferation and BRAFi resistance in melanoma [30,31]. Soluble mediators play a key role in the cross talk between tumor cells and TME. Therefore, to get further insight into the role of melanoma cell secretome in BRAFi resistance, we first evaluated the secretion of a set of cytokines/chemokines—known to be implicated in the modulation of immune responses and/or tumor cell proliferation and invasiveness—in melanoma cells sensitive or with acquired resistance to vemurafenib and assessed whether CM from these cells could affect DC maturation. We then carried out a multiplex analysis of CM of vemurafenib-sensitive and vemurafenib -resistant cells to identify additional soluble mediators potentially involved in BRAFi resistance.

As compared with parental cells, both vemurafenib-resistant cell clones showed in-creased secretion of IL-10, considered an important autocrine growth factor for malignant melanoma [32] and found to be upregulated in vemurafenib-resistant cells [33] of VEGF, a potent angiogenic factor also involved in BRAFi-resistance of melanoma cells [34], and of IL-1β and IL-8, cytokines that promote inflammation, tumorigenesis, and invasiveness [35]. Notably, increased expression of IL-8 has been found to be associated with multidrug resistance in breast cancer cells, sunitinib resistance in renal cell carcinoma, and RO4929097 (a γ-secretase inhibitor) resistance in NSCLC cells [36]. Upregulation of IL-8 secretion and MMP-2 activity was also reported by Sandri et al. [37] in melanoma cells with acquired resistance to vemurafenib, and our data are consistent with those findings and with the knowledge that MMP-2 activity is promoted by IL-8 expression [38]. On the other hand, a recent study by Hartman et al. [39] analyzing six different cell lines with acquired resistance to vemurafenib and their drug-sensitive counterparts evidenced a de-crease of IL-8 expression in four resistant cell lines and no changes in the other two cell lines. In the same study, a heterogenous phenotype of vemurafenib-resistant cells was also observed for MMP-2 expression, which was decreased in three cell lines, upregulated in one cell line, and not affected in the remaining two cell lines. Melanoma is a heterogenous tumor, and previous studies have shown that the transcriptional heterogeneity of melanoma cells affects their initial response to BRAFi and the development of resistance [40]. It is possible to hypothesize that the genetic background and the transcriptional heterogeneity at baseline of the melanoma cell lines used in the different studies to investigate mechanisms underlying acquired resistance to vemurafenib might be responsible for the diverse modulation of IL-8 and MMP-2 expression observed in the drug-resistant sublines. In the VR2 clone, we also detected enhanced secretion of the chemokines MCP-1, MIP-1α, MIP-1β, RANTES, and eotaxin, which play a key role in monocytes and DC chemotaxis [41]. Notably, MCP-1, also considered as autocrine growth factor for melanoma and a metastasis-inducer, is produced by BRAF-resistant melanoma cells, and its plasma level was shown to increase in melanoma patients during BRAFi treatments [42,43].

The presence of cytokines and chemokines able to induce DC maturation [41,44] prompted us to study the effects of melanoma CM on human DCs in order to extend the knowledge of the possible functional consequences of increased cytokine and chemokine secretion by vemurafenib-resistant melanoma cells. CM derived from the drug-resistant cells induced DC maturation, as was shown in the results of both phenotypic and cytokine/chemokine secretion analyses. Indeed, DCs exposed to CM from resistant cells showed increased expression of activation markers and released higher levels of pro-inflammatory factors, as was reported for IL-1β [35], than DCs treated with CMs from parental cells. Notably, IL-6 and MCP-1 were secreted at concentrations even higher than those observed in LPS-stimulated DCs. The interplay between melanoma and stromal cells in the TME, including DCs, is mainly supported by presence of IL-6, IL-10, and VEGF [13]. IL-6 promotes tumor growth by inhibition of apoptosis and induction of tumor angiogenesis. Increased serum concentration of IL-6 has been correlated with a worse prognosis in patients with melanoma, even if the specific biological functions of IL-6 in progression of melanoma are unknown [45]. It is noteworthy that the stimulation of DCs with CM from vemurafenib-resistant cells induced a significant release of IL-10, an immunosuppressive cytokine whose expression correlates with melanoma progression and metastasis [46]. It is well demonstrated, indeed, that melanoma alters DC in a pro-tumorigenic way and in a VEGF-dependent manner too [47,48]. Interestingly, the presence in melanoma CM of cytokines with antitumor activity, such as IL-12 and IFN-γ, has a potential role in DC maturation [49]. Accumulating evidence suggests that TME-located DCs, with intermediate mature states and expressing high levels of pro-inflammatory signals, might be considered as facilitators in cancer progression [50,51]. Our observations indicate for the first time that vemurafenib-resistant melanoma cells can modify DC maturation in order to benefit from their activation and subsequent cytokine production. Therefore, the delicate balance in the TME of cytokines levels may contribute, finally, to BRAFi resistance. Melanoma CM did not interfere with the upregulation of surface antigens on LPS-stimulated DCs (data not shown), suggesting their inability to interfere with DC under strong stimulation. In this study we investigated the effects of melanoma CM on DCs derived from monocytes of healthy donors. Of course, it is possible that in melanoma patients other factors may interfere with DC maturation, including the prevalence of other DC populations, such as the plasmacytoid or the CD34^+^ derived DCs, as well as a disabled maturation of monocytes into DC.

The mass spectrometry analysis identified 22 and 48 specific proteins in SK-MEL-28 and SK-MEL-28-VR2 cells, respectively. Enrichment analysis of DAVID (regarding the involved biological processes) highlighted that resistant cells suggest a higher activation of oxidative metabolism, in agreement with other studies [52,53]. Moreover, STRING analysis suggested a possible increase in the activity of the proteasome pathway in those cells. The proteasome is a large multi-subunit complex present in nucleus and cytoplasm that controls degradation of intracellular proteins. Due to this activity, the proteasome is strictly involved in many regulatory pathways within the tumor cell, including proliferation and apoptosis [24]. It should be noted that the presence of typical intracellular proteins (i.e., proteasome subunits) in CM was expected [54], since proteasome subunits occurrence in extracellular space and in plasma has been reported [55]. The proteasome role in BRAF-mutant cells has been widely explored. Recent studies have revealed that BRAF mutation enhances proteasome capacity and resistance to proteasome inhibitors in myeloma patients [56]. On the other hand, Zecchin and colleagues clearly demonstrated that proteasome inhibitors possess a significant selectivity toward BRAF^V600E^-mutant colorectal cancer cells as a consequence of persistent BRAF signaling and a nononcogenic addiction to the proteasome function in those cells [57]. Furthermore, the combination of bortezomib and vemurafenib was shown to produce synergistic antitumor effects in thyroid cancer, both in vitro and in a xenograft model [58]. In the present study, we show that our vemurafenib-resistant cells were more sensitive to bortezomib than parental cells. Based on the results of STRING analysis, we can hypothesize that the development of resistance to BRAFi renders the cells more dependent on proteasome function for proliferation and/or survival, leading to increased susceptibility to proteasome-targeting drugs. Further studies are however required to define the therapeutic potential of proteasome inhibitors in BRAFi-resistant melanomas since these agents have also been shown to have both immunosuppressive and immunostimulatory effects [59].

Bioinformatic analysis also indicated that vemurafenib-resistant cells expressed proteins related to cell adhesion and cell–cell and cell–matrix interactions. For example, we detected (Table 1) the presence of thrombospondin-1, which is involved in a melanoma epithelial-to-mesenchymal transition (EMT)-like process [60], or to protein FAM3C, and possibly related to EMT, tumor progression, and metastasis [61]. We also detected neuropilin-2, whose expression is known to be associated with melanoma progression [62]. However, we focused our attention on CD147/basigin since its expression was previously found to be upregulated in vemurafenib resistant cells [63]. CD147/basigin is a transmembrane protein member of the immunoglobulin superfamily that can shed from the cell membrane via an MMPs-dependent cleavage [64]. This soluble CD147 acts as a paracrine molecule able to stimulate the production of MMPs, with a consequent increase in the invasiveness of cancer cells [65]. The role of CD147/basigin in promoting melanoma proliferation, angiogenesis, progression, and metastasis is well documented [66]. In particular, downregulation of CD147/basigin induces apoptosis in melanoma cells [67] and impairs VEGF production [68]. Moreover, this molecule promotes tumor cell invasiveness by regulating MMP expression, including MMP-2, in neighboring fibroblasts or cancer cells [69], and previous studies have demonstrated that metalloproteases’ expression and enzymatic activity play an important role in determining an aggressive phenotype of melanoma cells [63]. As demonstrated by proteomic analyses and multiplex assays, VR2 and VR3 cells secreted a higher level of CD147/basigin than parental cells, a finding consistent with the enhanced secretion of VEGF and MMP-2 detected in the resistant cells [70] as well as with their predicted increased oxidative metabolism [71]. In this study, we demonstrated that in patients who developed resistance to vemurafenib or vemurafenib plus cobimetinib, plasma levels of CD147/basigin at disease progression were significantly higher than those detected before the start of therapy. These results, although preliminary and confirmed only in a small number of patients, suggest a possible contribution of CD147/basigin upregulation in BRAFi/MEKi resistance. Interestingly, several inhibitors [72] or an antibody [73] targeting CD147/basigin have been suggested as potential therapeutic agents.

## 5. Conclusions

Cell biology and proteomic studies carried out in the present study show that the interface between melanoma cells and TME, containing signals secreted by both cellular counterparts with autocrine and paracrine effects, represent a battlefield where specific signals may play a crucial role in BRAFi resistant cells. Therefore, the analysis of cancer secretome may provide useful tools and information to identify novel biomarkers and potential targets for new therapies [74]. Collectively, the observed increase of pro-inflammatory molecules, the overexpression of CD147/basigin, the reduction of adhesion ability, and the increase of MMP-2 secretions confirmed that our vemurafenib-resistant cells displayed a more disseminative phenotype compared to the parental one.

## Figures and Tables

**Table 1:** Specific (differentially expressed) proteins of parental and resistant cells.

Cells	Accession	Gene Symbol	Description
SK-MEL-28	P08195	SLC3A2	4F2 cell-surface antigen heavy chain
SK-MEL-28	Q9ULZ3	PYCARD	Apoptosis-associated speck-like protein containing a CARD
SK-MEL-28	P06865	HEXA	Beta-hexosaminidase subunit alpha
SK-MEL-28	P07711	CTSL	Cathepsin L1
SK-MEL-28	O00299	CLIC1	Chloride intracellular channel protein 1
SK-MEL-28	P63241	EIF5A	Eukaryotic translation initiation factor 5A-1
SK-MEL-28	P04439	HLA-A	HLA class I histocompatibility antigen, A-3 alpha chain
SK-MEL-28	P19013	KRT4	Keratin, type II cytoskeletal 4
SK-MEL-28	Q92859	NEO1	Neogenin
SK-MEL-28	Q02818	NUCB1	Nucleobindin-1
SK-MEL-28	P19338	NCL	Nucleolin
SK-MEL-28	P10451	SPP1	Osteopontin
SK-MEL-28	Q86UD1	OAF	out at first protein homolog
SK-MEL-28	Q6S8J3	POTEE	POTE ankyrin domain family member E
SK-MEL-28	Q15262	PTPRK	Receptor-type tyrosine-protein phosphatase kappa
SK-MEL-28	Q13332	PTPRS	Receptor-type tyrosine-protein phosphatase S
SK-MEL-28	Q12765	SCRN1	Secernin-1
SK-MEL-28	Q8N474	SFRP1	Secreted frizzled-related protein 1
SK-MEL-28	Q92743	HTRA1	Serine protease HTRA1
SK-MEL-28	P00441	SOD1	Superoxide dismutase [Cu-Zn]
SK-MEL-28	P78324	SIRPA	Tyrosine-protein phosphatase non-receptor type substrate 1
SK-MEL-28	P08670	VIM	Vimentin
SK-MEL-28-VR2	O14817	TSPAN4	Tetraspanin-4
SK-MEL-28-VR2	O43505	B3GNT1	Beta-1,4-glucuronyltransferase 1
SK-MEL-28-VR2	O60462	NRP2	Neuropilin-2
SK-MEL-28-VR2	O75874	IDH1	Isocitrate dehydrogenase
SK-MEL-28-VR2	P00338	LDHA	L-lactate dehydrogenase A chain
SK-MEL-28-VR2	P01130	LDLR	Low-density lipoprotein receptor
SK-MEL-28-VR2	P06276	BCHE	Cholinesterase
SK-MEL-28-VR2	P07237	P4HB	Protein disulfide-isomerase
SK-MEL-28-VR2	P32004	L1CAM	Neural cell adhesion molecule L1
SK-MEL-28-VR2	P35237	SERPINB6	Serpin B6
SK-MEL-28-VR2	P35613	BSG	Basigin
SK-MEL-28-VR2	P37802	TAGLN2	Transgelin-2
SK-MEL-28-VR2	P40967	PMEL	Melanocyte protein PMEL
SK-MEL-28-VR2	P42785	PRCP	Lysosomal Pro-X carboxypeptidase
SK-MEL-28-VR2	P46926	GNPDA1	Glucosamine-6-phosphate isomerase 1
SK-MEL-28-VR2	P48637	GSS	Glutathione synthetase
SK-MEL-28-VR2	P48745	NOV	Protein NOV homolog
SK-MEL-28-VR2	P49641	MAN2A2	Alpha-mannosidase 2x
SK-MEL-28-VR2	P52209	PGD	6-phosphogluconate dehydrogenase, decarboxylating
SK-MEL-28-VR2	P54819	AK2	Adenylate kinase 2, mitochondrial
SK-MEL-28-VR2	P16152	CBR1	Carbonyl reductase
SK-MEL-28-VR2	P16930	FAH	Fumarylacetoacetase
SK-MEL-28-VR2	P17174	GOT1	Aspartate aminotransferase, cytoplasmic
SK-MEL-28-VR2	P18669	PGAM1	Phosphoglycerate mutase 1
SK-MEL-28-VR2	P19021	PAM	Peptidyl-glycine alpha-amidating monooxygenase
SK-MEL-28-VR2	P20618	PSMB1	Proteasome subunit beta type-1
SK-MEL-28-VR2	P22392	NME1	Nucleoside diphosphate kinase B
SK-MEL-28-VR2	P25787	PSMA2	Proteasome subunit alpha type-2
SK-MEL-28-VR2	P12955	PEPD	Xaa-Pro dipeptidase
SK-MEL-28-VR2	P07686	HEXB	Beta-hexosaminidase subunit beta
SK-MEL-28-VR2	P07996	THBS1	Thrombospondin-1
SK-MEL-28-VR2	P99999	CYCS	Cytochrome c
SK-MEL-28-VR2	Q01469	FABP5	Fatty acid-binding protein, epidermal
SK-MEL-28-VR2	Q96KP4	CNDP2	Cytosolic non-specific dipeptidase
SK-MEL-28-VR2	Q9H2E6	SEMA6A	Semaphorin-6A
SK-MEL-28-VR2	Q9NPF2	CHST11	Carbohydrate sulfotransferase 11
SK-MEL-28-VR2	Q9P2B2	PTGFRN	Prostaglandin F2 receptor negative regulator
SK-MEL-28-VR2	Q9UBQ6	EXTL2	Exostosin-like 2
SK-MEL-28-VR2	Q9UNW1	MINPP1	Multiple inositol polyphosphate phosphatase 1
SK-MEL-28-VR2	Q9Y281	CFL2	Cofilin-2
SK-MEL-28-VR2	Q6IBS0	TWF2	Twinfilin-2
SK-MEL-28-VR2	Q8NBJ7	SUMF2	Sulfatase-modifying factor 2
SK-MEL-28-VR2	Q8NCW5	APOA1BP	NAD(P)H-hydrate epimerase
SK-MEL-28-VR2	Q8WVQ1	CANT1	Soluble calcium-activated nucleotidase 1
SK-MEL-28-VR2	Q92520	FAM3C	Protein FAM3C
SK-MEL-28-VR2	Q06830	PRDX1	Peroxiredoxin-1
SK-MEL-28-VR2	Q14019	COTL1	Coactosin-like protein
SK-MEL-28-VR2	Q15084	PDIA6	Protein disulfide-isomerase A6

**Table 2:** Demographic and clinical characteristics of melanoma patients from whom plasma was collected.

Case	Sex	Age (Years)	Stage ^a^	LDH ^b^	Previous Therapy	Targeted Therapy ^c^	BR ^d^	TTF ^e^ (Days)
Pt#1	M	57	M1c	H	None	VEMU	PR	223
Pt#2	M	49	M1c	H	None	VEMU	PR	147
Pt#3	F	74	M1b	N	None	VEMU	PR	195
Pt#4	M	50	M1a	N	None	VEMU + COBI	PR	362
Pt#5	M	58	M1a	H	None	VEMU + COBI	PR	195

^a^ Stage at first plasma collection (i.e., T0). ^b^ LDH, lactate dehydrogenase; N, normal; H, >1.5 upper limit of normal values. ^c^ VEMU, vemurafenib, COBI, cobimetinib. ^d^ Best response according to RECIST 1.1 criteria: PR, partial response. ^e^ TTF, time-to-treatment-failure.

## Data Availability

Data available on request by contacting either of the corresponding authors depending on privacy/ethical restrictions.

## Supplementary Materials

The following are available online at https://www.mdpi.com/2227-9059/9/1/79/s1: Figure S1, Vemurafenib-sensitivity of SK-MEL-28 parental and resistant cells; Table S1, Phenotypic changes of dendritic cells (DC) cultured with conditioned medium of melanoma cells; and Table S2, List of proteins identified by proteomic analysis.
