# Supplementary material for: Melanoma Cell Resistance to Vemurafenib Modifies Inter-Cellular Communication Signals
Source: Biomedicines. 2021 Jan 15;9(1):79. doi: 10.3390/biomedicines9010079 (PMC7830125; doi:10.3390/biomedicines9010079)
Supplement: Supplementary file 1 [file biomedicines-09-00079-s001.pdf]

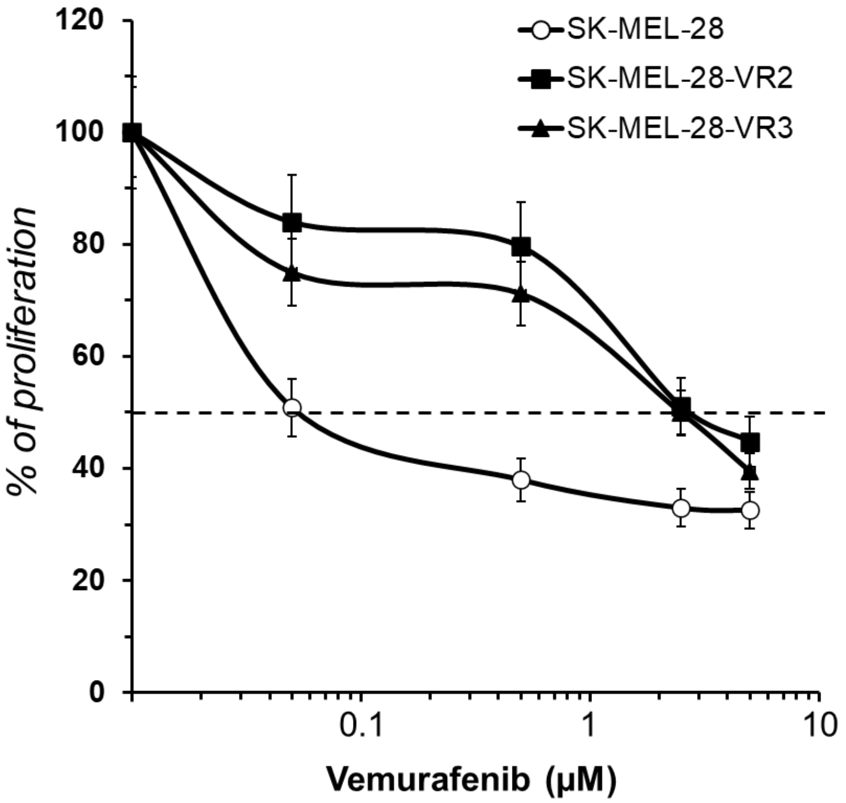

Figure S1. Vemurafenib-sensitivity of SK-MEL-28 parental and resistant cells. Cell proliferation at in increasing concentrations of vemurafenib were determined by sulphorhodamine B (SRB) assay (72 h). Resistant clones were cultured for one cell cycle in the absence of vemurafenib before each experiment. The dotted line indicates the half-maximal effect. Error bars represent the standard deviation.

Table S1
Phenotypic changes of dendritic cells (DC) cultured with conditioned medium of melanoma cells.

	Relative increase in surface expression*				
	CD80	CD86	CD83	CLASS I	CLASS II
LPS	5.35	5.75	3.12	1.63	1.72
SK-MEL-28	1.94	1.61	1.58	0.97	1.09
SK-MEL-28-VR2	2.59	3.46	2.56	0.98	1.57
SK-MEL-28-VR3	2.27	2.47	2.09	0.92	1.23

* The relative increase of indicated surface markers is expressed dividing the median fluorescence intensity (MFI) by the value of the corresponding markers for DC in medium alone (Control). The values of MFI for control DCs, in this representative assay, were 4.2, 12.4, 10.7, 10.9, and 31.5 for CD80, CB86, CD83, CLASS I, and CLASS II, respectively.

Table S2

List of proteins identified by proteomic analysis.

	Cells	Accession	Description	MW [kDa]
1	SK-MEL-28	**P08195**	4F2 cell-surface antigen heavy chain	68.0
2	SK-MEL-28	**P62258**	14-3-3 protein epsilon	29.2
3	SK-MEL-28	**P63104**	14-3-3 protein zeta/delta	27.7
4	SK-MEL-28	**P68032**	Actin, alpha cardiac muscle 1	42.0
5	SK-MEL-28	**P60709**	Actin, cytoplasmic 1	41.7
6	SK-MEL-28	**O00468**	Agrin	217.1
7	SK-MEL-28	**P14550**	Alcohol dehydrogenase [NADP(+)]	36.5
8	SK-MEL-28	**P01023**	Alpha-2-macroglobulin	163.2
9	SK-MEL-28	**P12814**	Alpha-actinin-1	103.0
10	SK-MEL-28	**O43707**	Alpha-actinin-4	104.8
11	SK-MEL-28	**P06733**	Alpha-enolase	47.1
12	SK-MEL-28	**P05067**	Amyloid beta A4 protein	86.9
13	SK-MEL-28	**P04083**	Annexin A1	38.7
14	SK-MEL-28	**P07355**	Annexin A2	38.6
15	SK-MEL-28	**P08758**	Annexin A5	35.9
16	SK-MEL-28	**P01008**	Antithrombin-III	52.6
17	SK-MEL-28	**P02649**	Apolipoprotein E	36.1
18	SK-MEL-28	**Q9ULZ3**	Apoptosis-associated speck-like protein containing a CARD	21.6
19	SK-MEL-28	**P98160**	Basement membrane-specific heparan sulfate proteoglycan core protein	468.5
20	SK-MEL-28	**P06865**	Beta-hexosaminidase subunit alpha	60.7
21	SK-MEL-28	**P27797**	Calreticulin	48.1
22	SK-MEL-28	**O94985**	Calsyntenin-1	109.7
23	SK-MEL-28	**O43852**	Calumenin	37.1

24	SK-MEL-28	**P07858**	Cathepsin B	37.8
25	SK-MEL-28	**P07339**	Cathepsin D	44.5
26	SK-MEL-28	**P07711**	Cathepsin L1	37.5
27	SK-MEL-28	**Q9UBR2**	Cathepsin Z	33.8
28	SK-MEL-28	**P11717**	Cation-independent mannose-6-phosphate receptor	274.2
29	SK-MEL-28	**Q6YHK3**	CD109 antigen	161.6
30	SK-MEL-28	**P60033**	CD81 antigen	25.8
31	SK-MEL-28	**P43121**	Cell surface glycoprotein MUC18	71.6
32	SK-MEL-28	**Q9BWS9**	Chitinase domain-containing protein 1	44.9
33	SK-MEL-28	**O00299**	Chloride intracellular channel protein 1	26.9
34	SK-MEL-28	**Q6UVK1**	Chondroitin sulfate proteoglycan 4	250.4
35	SK-MEL-28	**P23528**	Cofilin-1	18.5
36	SK-MEL-28	**Q96CG8**	Collagen triple helix repeat-containing protein 1	26.2
37	SK-MEL-28	**Q9ULV4**	Coronin-1C	53.2
38	SK-MEL-28	**Q14118**	Dystroglycan	97.4
39	SK-MEL-28	**Q9UNN8**	Endothelial protein C receptor	26.7
40	SK-MEL-28	**P63241**	Eukaryotic translation initiation factor 5A-1	16.8
41	SK-MEL-28	**Q16610**	Extracellular matrix protein 1	60.6
42	SK-MEL-28	**P02751**	Fibronectin	262.5
43	SK-MEL-28	**Q12841**	Follistatin-related protein 1	35.0
44	SK-MEL-28	**P04075**	Fructose-bisphosphate aldolase A	39.4
45	SK-MEL-28	**P09972**	Fructose-bisphosphate aldolase C	39.4
46	SK-MEL-28	**Q08380**	Galectin-3-binding protein	65.3
47	SK-MEL-28	**P09104**	Gamma-enolase	47.2
48	SK-MEL-28	**Q92820**	Gamma-glutamyl hydrolase	35.9
49	SK-MEL-28	**P06396**	Gelsolin	85.6
50	SK-MEL-28	**P07093**	Glia-derived nexin	44.0
51	SK-MEL-28	**P06744**	Glucose-6-phosphate isomerase	63.1
52	SK-MEL-28	**Q16769**	Glutaminyl-peptide cyclotransferase	40.9
53	SK-MEL-28	**P09211**	Glutathione S-transferase P	23.3

54	SK-MEL-28	**P04406**	Glyceraldehyde-3-phosphate dehydrogenase	36.0
55	SK-MEL-28	**P34932**	Heat shock 70 kDa protein 4	94.3
56	SK-MEL-28	**P06899**	Histone H2B type 1-J	13.9
57	SK-MEL-28	**P04439**	HLA class I histocompatibility antigen, A-3 alpha chain	40.8
58	SK-MEL-28	**Q16270**	Insulin-like growth factor-binding protein 7	29.1
59	SK-MEL-28	**P05362**	Intercellular adhesion molecule 1	57.8
60	SK-MEL-28	**P13645**	Keratin, type I cytoskeletal 10	58.8
61	SK-MEL-28	**P02533**	Keratin, type I cytoskeletal 14	51.5
62	SK-MEL-28	**P08779**	Keratin, type I cytoskeletal 16	51.2
63	SK-MEL-28	**P35527**	Keratin, type I cytoskeletal 9	62.0
64	SK-MEL-28	**P04264**	Keratin, type II cytoskeletal 1	66.0
65	SK-MEL-28	**P35908**	Keratin, type II cytoskeletal 2 epidermal	65.4
66	SK-MEL-28	**P19013**	Keratin, type II cytoskeletal 4	57.2
67	SK-MEL-28	**P13647**	Keratin, type II cytoskeletal 5	62.3
68	SK-MEL-28	**P02538**	Keratin, type II cytoskeletal 6A	60.0
69	SK-MEL-28	**Q08431**	Lactadherin	43.1
70	SK-MEL-28	**P25391**	Laminin subunit alpha-1	336.9
71	SK-MEL-28	**Q16363**	Laminin subunit alpha-4	202.4
72	SK-MEL-28	**P07942**	Laminin subunit beta-1	197.9
73	SK-MEL-28	**P55268**	Laminin subunit beta-2	195.9
74	SK-MEL-28	**P11047**	Laminin subunit gamma-1	177.5
75	SK-MEL-28	**P07195**	L-lactate dehydrogenase B chain	36.6
76	SK-MEL-28	**P40121**	Macrophage-capping protein	38.5
77	SK-MEL-28	**P40925**	Malate dehydrogenase, cytoplasmic	36.4
78	SK-MEL-28	**Q16674**	Melanoma-derived growth regulatory protein	14.5
79	SK-MEL-28	**P08582**	Melanotransferrin O	80.2
80	SK-MEL-28	**P16035**	Metalloproteinase inhibitor 2	24.4
81	SK-MEL-28	**P26038**	Moesin	67.8
82	SK-MEL-28	**Q92859**	Neogenin	159.9
83	SK-MEL-28	**Q92823**	Neuronal cell adhesion molecule	143.8

84	SK-MEL-28	**O15240**	Neurosecretory protein VGF	67.2
85	SK-MEL-28	**P43007**	Neutral amino acid transporter A	55.7
86	SK-MEL-28	**P22894**	Neutrophil collagenase	53.4
87	SK-MEL-28	**Q02818**	Nucleobindin-1	53.8
88	SK-MEL-28	**P19338**	Nucleolin	76.6
89	SK-MEL-28	**P06748**	Nucleophosmin	32.6
90	SK-MEL-28	**P10451**	Osteopontin	35.4
91	SK-MEL-28	**Q86UD1**	out at first protein homolog	30.7
92	SK-MEL-28	**P62937**	Peptidyl-prolyl cis-trans isomerase A	18.0
93	SK-MEL-28	**P23284**	Peptidyl-prolyl cis-trans isomerase B	23.7
94	SK-MEL-28	**P30086**	Phosphatidylethanolamine-binding protein 1	21.0
95	SK-MEL-28	**P00558**	Phosphoglycerate kinase 1	44.6
96	SK-MEL-28	**Q10471**	Polypeptide N-acetylgalactosaminyltransferase 2	64.7
97	SK-MEL-28	**Q6S8J3**	POTE ankyrin domain family member E	121.3
98	SK-MEL-28	**P20742**	Pregnancy zone protein	163.8
99	SK-MEL-28	**P09668**	Pro-cathepsin H	37.4
100	SK-MEL-28	**Q02809**	Procollagen-lysine,2-oxoglutarate 5-dioxygenase 1	83.5
101	SK-MEL-28	**O60568**	Procollagen-lysine,2-oxoglutarate 5-dioxygenase 3	84.7
102	SK-MEL-28	**P07737**	Profilin-1	15.0
103	SK-MEL-28	**Q8WUM4**	Programmed cell death 6-interacting protein	96.0
104	SK-MEL-28	**P30101**	Protein disulfide-isomerase A3	56.7
105	SK-MEL-28	**P14618**	Pyruvate kinase PKM	57.9
106	SK-MEL-28	**P50395**	Rab GDP dissociation inhibitor beta	50.6
107	SK-MEL-28	**Q15262**	Receptor-type tyrosine-protein phosphatase kappa	162.0
108	SK-MEL-28	**Q13332**	Receptor-type tyrosine-protein phosphatase S	216.9
109	SK-MEL-28	**Q12765**	Secernin-1	46.4
110	SK-MEL-28	**Q8N474**	Secreted frizzled-related protein 1	35.4
111	SK-MEL-28	**Q92743**	Serine protease HTRA1	51.3
112	SK-MEL-28	**P02768**	Serum albumin	69.3
113	SK-MEL-28	**P09486**	SPARC	34.6

114	SK-MEL-28	**Q9BUD6**	Spondin-2	35.8
115	SK-MEL-28	**O00391**	Sulfhydryl oxidase 1	82.5
116	SK-MEL-28	**P00441**	Superoxide dismutase [Cu-Zn]	15.9
117	SK-MEL-28	**P78539**	Sushi repeat-containing protein SRPX	51.5
118	SK-MEL-28	**O00560**	Syntenin-1	32.4
119	SK-MEL-28	**P13686**	Tartrate-resistant acid phosphatase type 5	36.6
120	SK-MEL-28	**P24821**	Tenascin	240.7
121	SK-MEL-28	**Q16881**	Thioredoxin reductase 1, cytoplasmic	70.9
122	SK-MEL-28	**P55072**	Transitional endoplasmic reticulum ATPase	89.3
123	SK-MEL-28	**P29401**	Transketolase	67.8
124	SK-MEL-28	**P60174**	Triosephosphate isomerase	30.8
125	SK-MEL-28	**P78324**	Tyrosine-protein phosphatase non-receptor type substrate 1	54.9
126	SK-MEL-28	**Q12907**	Vesicular integral-membrane protein VIP36	40.2
127	SK-MEL-28	**P08670**	Vimentin	53.6
128	SK-MEL-28	**P02774**	Vitamin D-binding protein	52.9
129	SK-MEL-28	**P07225**	Vitamin K-dependent protein S	75.1
1	SK-MEL-28-VR2	**P62258**	14-3-3 protein epsilon	29.2
2	SK-MEL-28-VR2	**P63104**	14-3-3 protein zeta/delta	27.7
3	SK-MEL-28-VR2	**P52209**	6-phosphogluconate dehydrogenase, decarboxylating	53.1
4	SK-MEL-28-VR2	**P68032**	Actin, alpha cardiac muscle 1	42.0
5	SK-MEL-28-VR2	**P60709**	Actin, cytoplasmic 1	41.7
6	SK-MEL-28-VR2	**P54819**	Adenylate kinase 2, mitochondrial	26.5
7	SK-MEL-28-VR2	**O00468**	Agrin	217.1
8	SK-MEL-28-VR2	**P14550**	Alcohol dehydrogenase	36.5
9	SK-MEL-28-VR2	**P01023**	Alpha-2-macroglobulin	163.2
10	SK-MEL-28-VR2	**P12814**	Alpha-actinin-1	103.0
11	SK-MEL-28-VR2	**O43707**	Alpha-actinin-4	104.8
12	SK-MEL-28-VR2	**P06733**	Alpha-enolase	47.1
13	SK-MEL-28-VR2	**P49641**	Alpha-mannosidase 2x	130.5

14	SK-MEL-28-VR2	**P05067**	Amyloid beta A4 protein	86.9
15	SK-MEL-28-VR2	**P04083**	Annexin A1	38.7
16	SK-MEL-28-VR2	**P07355**	Annexin A2	38.6
17	SK-MEL-28-VR2	**P08758**	Annexin A5	35.9
18	SK-MEL-28-VR2	**P01008**	Antithrombin-III	52.6
19	SK-MEL-28-VR2	**P02649**	Apolipoprotein E	36.1
20	SK-MEL-28-VR2	**P17174**	Aspartate aminotransferase, cytoplasmic	46.2
21	SK-MEL-28-VR2	**P98160**	Basement membrane-specific heparan sulfate proteoglycan core protein	468.5
22	SK-MEL-28-VR2	**P35613**	Basigin	42.2
23	SK-MEL-28-VR2	**O43505**	Beta-1,4-glucuronyltransferase 1	47.1
24	SK-MEL-28-VR2	**P07686**	Beta-hexosaminidase subunit beta	63.1
25	SK-MEL-28-VR2	**P27797**	Calreticulin	48.1
26	SK-MEL-28-VR2	**O94985**	Calsyntenin-1	109.7
27	SK-MEL-28-VR2	**O43852**	Calumenin	37.1
28	SK-MEL-28-VR2	**Q9NPF2**	Carbohydrate sulfotransferase 11	41.5
29	SK-MEL-28-VR2	**P16152**	Carbonyl reductase	30.4
30	SK-MEL-28-VR2	**P07858**	Cathepsin B	37.8
31	SK-MEL-28-VR2	**P07339**	Cathepsin D	44.5
32	SK-MEL-28-VR2	**Q9UBR2**	Cathepsin Z	33.8
33	SK-MEL-28-VR2	**P11717**	Cation-independent mannose-6-phosphate receptor	274.2
34	SK-MEL-28-VR2	**Q6YHK3**	CD109 antigen	161.6
35	SK-MEL-28-VR2	**P60033**	CD81 antigen	25.8
36	SK-MEL-28-VR2	**P43121**	Cell surface glycoprotein MUC18	71.6
37	SK-MEL-28-VR2	**Q9BWS9**	Chitinase domain-containing protein 1	44.9
38	SK-MEL-28-VR2	**P06276**	Cholinesterase	68.4
39	SK-MEL-28-VR2	**Q6UVK1**	Chondroitin sulfate proteoglycan 4	250.4
40	SK-MEL-28-VR2	**Q14019**	Coactosin-like protein	15.9
41	SK-MEL-28-VR2	**P23528**	Cofilin-1	18.5
42	SK-MEL-28-VR2	**Q9Y281**	Cofilin-2	18.7

43	SK-MEL-28-VR2	**Q96CG8**	Collagen triple helix repeat-containing protein 1	26.2
44	SK-MEL-28-VR2	**Q9ULV4**	Coronin-1C	53.2
45	SK-MEL-28-VR2	**P99999**	Cytochrome c	11.7
46	SK-MEL-28-VR2	**Q96KP4**	Cytosolic non-specific dipeptidase	52.8
47	SK-MEL-28-VR2	**Q14118**	Dystroglycan	97.4
48	SK-MEL-28-VR2	**Q9UNN8**	Endothelial protein C receptor	26.7
49	SK-MEL-28-VR2	**Q9UBQ6**	Exostosin-like 2	37.4
50	SK-MEL-28-VR2	**Q16610**	Extracellular matrix protein 1	60.6
51	SK-MEL-28-VR2	**Q01469**	Fatty acid-binding protein, epidermal	15.2
52	SK-MEL-28-VR2	**P02751**	Fibronectin	262.5
53	SK-MEL-28-VR2	**Q12841**	Follistatin-related protein 1	35.0
54	SK-MEL-28-VR2	**P04075**	Fructose-bisphosphate aldolase A	39.4
55	SK-MEL-28-VR2	**P09972**	Fructose-bisphosphate aldolase C	39.4
56	SK-MEL-28-VR2	**P16930**	Fumarylacetoacetase	46.3
57	SK-MEL-28-VR2	**Q08380**	Galectin-3-binding protein	65.3
58	SK-MEL-28-VR2	**P09104**	Gamma-enolase	47.2
59	SK-MEL-28-VR2	**Q92820**	Gamma-glutamyl hydrolase	35.9
60	SK-MEL-28-VR2	**P06396**	Gelsolin	85.6
61	SK-MEL-28-VR2	**P07093**	Glia-derived nexin	44.0
62	SK-MEL-28-VR2	**P46926**	Glucosamine-6-phosphate isomerase 1	32.6
63	SK-MEL-28-VR2	**P06744**	Glucose-6-phosphate isomerase	63.1
64	SK-MEL-28-VR2	**Q16769**	Glutaminyl-peptide cyclotransferase	40.9
65	SK-MEL-28-VR2	**P09211**	Glutathione S-transferase P	23.3
66	SK-MEL-28-VR2	**P48637**	Glutathione synthetase	52.4
67	SK-MEL-28-VR2	**P04406**	Glyceraldehyde-3-phosphate dehydrogenase	36.0
68	SK-MEL-28-VR2	**P34932**	Heat shock 70 kDa protein 4	94.3
69	SK-MEL-28-VR2	**P06899**	Histone H2B type 1-J	13.9
70	SK-MEL-28-VR2	**Q16270**	Insulin-like growth factor-binding protein 7	29.1
71	SK-MEL-28-VR2	**P05362**	Intercellular adhesion molecule 1	57.8
72	SK-MEL-28-VR2	**O75874**	Isocitrate dehydrogenase	46.6

73	SK-MEL-28-VR2	**P13645**	Keratin, type I cytoskeletal 10	58.8
74	SK-MEL-28-VR2	**P02533**	Keratin, type I cytoskeletal 14	51.5
75	SK-MEL-28-VR2	**P08779**	Keratin, type I cytoskeletal 16	51.2
76	SK-MEL-28-VR2	**P35527**	Keratin, type I cytoskeletal 9	62.0
77	SK-MEL-28-VR2	**P04264**	Keratin, type II cytoskeletal 1	66.0
78	SK-MEL-28-VR2	**P35908**	Keratin, type II cytoskeletal 2 epidermal	65.4
79	SK-MEL-28-VR2	**P13647**	Keratin, type II cytoskeletal 5	62.3
80	SK-MEL-28-VR2	**P02538**	Keratin, type II cytoskeletal 6A	60.0
81	SK-MEL-28-VR2	**Q08431**	Lactadherin	43.1
82	SK-MEL-28-VR2	**P25391**	Laminin subunit alpha-1	336.9
83	SK-MEL-28-VR2	**Q16363**	Laminin subunit alpha-4	202.4
84	SK-MEL-28-VR2	**P07942**	Laminin subunit beta-1	197.9
85	SK-MEL-28-VR2	**P55268**	Laminin subunit beta-2	195.9
86	SK-MEL-28-VR2	**P11047**	Laminin subunit gamma-1	177.5
87	SK-MEL-28-VR2	**P00338**	L-lactate dehydrogenase A chain	36.7
88	SK-MEL-28-VR2	**P07195**	L-lactate dehydrogenase B chain	36.6
89	SK-MEL-28-VR2	**P01130**	Low-density lipoprotein receptor	95.3
90	SK-MEL-28-VR2	**P42785**	Lysosomal Pro-X carboxypeptidase	55.8
91	SK-MEL-28-VR2	**P40121**	Macrophage-capping protein	38.5
92	SK-MEL-28-VR2	**P40925**	Malate dehydrogenase, cytoplasmic	36.4
93	SK-MEL-28-VR2	**P40967**	Melanocyte protein PMEL	70.2
94	SK-MEL-28-VR2	**Q16674**	Melanoma-derived growth regulatory protein	14.5
95	SK-MEL-28-VR2	**P08582**	Melanotransferrin	80.2
96	SK-MEL-28-VR2	**P16035**	Metalloproteinase inhibitor 2	24.4
97	SK-MEL-28-VR2	**P26038**	Moesin	67.8
98	SK-MEL-28-VR2	**Q9UNW1**	Multiple inositol polyphosphate phosphatase 1	55.0
99	SK-MEL-28-VR2	**Q8NCW5**	NAD(P)H-hydrate epimerase	31.7
100	SK-MEL-28-VR2	**P32004**	Neural cell adhesion molecule L1	139.9
101	SK-MEL-28-VR2	**Q92823**	Neuronal cell adhesion molecule	143.8
102	SK-MEL-28-VR2	**O60462**	Neuropilin-2	104.8

103	SK-MEL-28-VR2	**O15240**	Neurosecretory protein VGF	67.2
104	SK-MEL-28-VR2	**P43007**	Neutral amino acid transporter A	55.7
105	SK-MEL-28-VR2	**P22894**	Neutrophil collagenase	53.4
106	SK-MEL-28-VR2	**P06748**	Nucleophosmin	32.6
107	SK-MEL-28-VR2	**P22392**	Nucleoside diphosphate kinase B	17.3
108	SK-MEL-28-VR2	**P19021**	Peptidyl-glycine alpha-amidating monooxygenase	108.3
109	SK-MEL-28-VR2	**P62937**	Peptidyl-prolyl cis-trans isomerase A	18.0
110	SK-MEL-28-VR2	**P23284**	Peptidyl-prolyl cis-trans isomerase B	23.7
111	SK-MEL-28-VR2	**Q06830**	Peroxiredoxin-1	22.1
112	SK-MEL-28-VR2	**P30086**	Phosphatidylethanolamine-binding protein 1	21.0
113	SK-MEL-28-VR2	**P00558**	Phosphoglycerate kinase 1	44.6
114	SK-MEL-28-VR2	**P18669**	Phosphoglycerate mutase 1	28.8
115	SK-MEL-28-VR2	**Q10471**	Polypeptide N-acetylgalactosaminyltransferase 2	64.7
116	SK-MEL-28-VR2	**P20742**	Pregnancy zone protein	163.8
117	SK-MEL-28-VR2	**P09668**	Pro-cathepsin H	37.4
118	SK-MEL-28-VR2	**Q02809**	Procollagen-lysine,2-oxoglutarate 5-dioxygenase 1	83.5
119	SK-MEL-28-VR2	**O60568**	Procollagen-lysine,2-oxoglutarate 5-dioxygenase 3	84.7
120	SK-MEL-28-VR2	**P07737**	Profilin-1	15.0
121	SK-MEL-28-VR2	**Q8WUM4**	Programmed cell death 6-interacting protein	96.0
122	SK-MEL-28-VR2	**Q9P2B2**	Prostaglandin F2 receptor negative regulator	98.5
123	SK-MEL-28-VR2	**P25787**	Proteasome subunit alpha type-2	25.9
124	SK-MEL-28-VR2	**P20618**	Proteasome subunit beta type-1	26.5
125	SK-MEL-28-VR2	**P07237**	Protein disulfide-isomerase	57.1
126	SK-MEL-28-VR2	**P30101**	Protein disulfide-isomerase A3	56.7
127	SK-MEL-28-VR2	**Q15084**	Protein disulfide-isomerase A6	48.1
128	SK-MEL-28-VR2	**Q92520**	Protein FAM3C	24.7
129	SK-MEL-28-VR2	**P48745**	Protein NOV homolog	39.1
130	SK-MEL-28-VR2	**P14618**	Pyruvate kinase PKM	57.9
131	SK-MEL-28-VR2	**P50395**	Rab GDP dissociation inhibitor beta	50.6
132	SK-MEL-28-VR2	**Q9H2E6**	Semaphorin-6A	114.3

133	SK-MEL-28-VR2	**P35237**	Serpin B6	42.6
134	SK-MEL-28-VR2	**P02768**	Serum albumin	69.3
135	SK-MEL-28-VR2	**Q8WVQ1**	Soluble calcium-activated nucleotidase 1	44.8
136	SK-MEL-28-VR2	**P09486**	SPARC	34.6
137	SK-MEL-28-VR2	**Q9BUD6**	Spondin-2	35.8
138	SK-MEL-28-VR2	**Q8NBJ7**	Sulfatase-modifying factor 2	33.8
139	SK-MEL-28-VR2	**O00391**	Sulfhydryl oxidase 1	82.5
140	SK-MEL-28-VR2	**P78539**	Sushi repeat-containing protein SRPX	51.5
141	SK-MEL-28-VR2	**O00560**	Syntenin-1	32.4
142	SK-MEL-28-VR2	**P13686**	Tartrate-resistant acid phosphatase type 5	36.6
143	SK-MEL-28-VR2	**P24821**	Tenascin	240.7
144	SK-MEL-28-VR2	**O14817**	Tetraspanin-4	26.1
145	SK-MEL-28-VR2	**Q16881**	Thioredoxin reductase 1, cytoplasmic	70.9
146	SK-MEL-28-VR2	**P07996**	Thrombospondin-1	129.3
147	SK-MEL-28-VR2	**P37802**	Transgelin-2	22.4
148	SK-MEL-28-VR2	**P55072**	Transitional endoplasmic reticulum ATPase	89.3
149	SK-MEL-28-VR2	**P29401**	Transketolase	67.8
150	SK-MEL-28-VR2	**P60174**	Triosephosphate isomerase	30.8
151	SK-MEL-28-VR2	**Q6IBS0**	Twinfilin-2	39.5
152	SK-MEL-28-VR2	**Q12907**	Vesicular integral-membrane protein VIP36	40.2
153	SK-MEL-28-VR2	**P02774**	Vitamin D-binding protein	52.9
154	SK-MEL-28-VR2	**P07225**	Vitamin K-dependent protein S	75.1
155	SK-MEL-28-VR2	**P12955**	Xaa-Pro dipeptidase	54.5
